# Differences in Treatment Modalities and Prognosis of Elderly Patients with Ovarian Cancer: A Two-Center Propensity Score-Matched Study

**DOI:** 10.3390/cancers14153655

**Published:** 2022-07-27

**Authors:** Yuxi Zhao, Jing Zuo, Ning Li, Rongshou Zheng, Guangwen Yuan, Guihua Shen, Lingying Wu

**Affiliations:** 1 Department of Gynecologic Oncology, National Cancer Center/National Clinical Research Center for Cancer/Cancer Hospital, Chinese Academy of Medical Science and Peking Union Medical College, Beijing 100021, China; drzhaoyuxi@163.com (Y.Z.); zuojing01894@163.com (J.Z.); liningnci@126.com (N.L.); william327@126.com (G.Y.); 2 Office of National Central Cancer Registry, National Cancer Center/National Clinical Research Center for Cancer/Cancer Hospital, Chinese Academy of Medical Science and Peking Union Medical College, Beijing 100021, China; zhengrongshou@163.com; 3 Department of Obstetrics and Gynecology, National Center of Gerontology/Beijing Hospital, Beijing 100730, China

**Keywords:** ovarian cancer, older patient, prognosis, adjuvant chemotherapy, albumin

## Abstract

**Simple Summary:**

The standard of care for patients with ovarian cancer is well established. While the prognosis of younger patients has improved significantly, the survival of older patients remains low. Reasons accounting for the poor survival among older patients are controversial. This study aimed to describe the difference in treatment received by older patients in China and to evaluate the prognostic significance of both chronological age and different treatment modalities using a propensity score-matched cohort. Cytoreduction with no residual tumor and the completion of adjuvant chemotherapy were the most important prognostic factors. Chronological age, however, had no influence on recurrence. This relatively large case–controlled study provides evidence that the difference between the standard of care and the treatment of older patients was the main reason that handicaps the efficacy of therapy.

**Abstract:**

Background: The prognosis of older patients with ovarian cancer is poor. We evaluated the effect of chronological age and different treatment characteristics on the prognosis of older patients with ovarian cancer; Methods: The study retrospectively analyzed patients aged over 60 years who underwent cytoreduction followed by platinum-based adjuvant chemotherapy between January 2011 and December 2019 in two national centers in China. Propensity score matching (PSM, 1:1) was performed to stratify the comorbidity- and treatment-related factors. The Kaplan–Meier method was employed to estimate progression-free survival (PFS) in the original cohort and the cohort after PSM; Results: A total of 324 patients were evaluated. The Age ≥ 70 group often received more neoadjuvant chemotherapy (62.3% vs. 31.2%, *p* < 0.001), more discontinuation of adjuvant chemotherapy (31.2% vs. 10.8%, *p* < 0.001), and had more severe chemotherapy-related toxicity (45.6% vs. 34.2%, *p* = 0.040) than the Age < 70 group. After matching, the PFS of the Age < 70 group was not significantly different from the Age ≥ 70 group (median PFS = 12.4 and 11.9 months, respectively, *p* = 0.850). Furthermore, the advanced FIGO stage, non-R0 cytoreduction, and discontinuation of adjuvant chemotherapy were all found to be poor prognostic factors. Serum albumin level <40 g/L (HR = 2.441, *p* = 0.018) and age ≥ 70 years (HR = 2.639, *p* = 0.008) led to more severe chemotherapy-related toxicity. Additionally, poor renal function (HR = 5.128, *p* = 0.002) was in association with discontinuation of adjuvant chemotherapy; Conclusions: The chronological age of older patients cannot be seen as a poor prognostic factor. Older patients may benefit most from R0 cytoreduction followed by the completion of chemotherapy. Postoperative poor renal function and serum albumin level <40 g/L may help predict the discontinuation of adjuvant chemotherapy.

## 1. Introduction

Ovarian cancer (OC) is the leading cause of death among gynecologic malignancies, with a median age of 63 years at diagnosis [1]. In 2016, there were approximately 57,200 new ovarian cancer cases and 27,200 deaths related to this disease in China [2]. Older patients with cancer have a poorer prognosis. Patients aged >70 years account for more than 80% of the death population [3].

The cornerstone of OC treatment includes cytoreduction followed by platinum-based adjuvant chemotherapy [4]. The prognosis of patients has improved since adopting the standard of care, as continuous improvement in prognosis was witnessed in the younger cohort, yet the survival of the older cohort has remained unchanged since 1979 [5]. Today, the mortality of patients aged over 70 years is 16-times higher than that of those aged less than 65 years [6].

Chronological age has long been considered as a poor prognostic factor [7]. Two cohort-based studies suggested that age >70 years was an independent risk factor for high mortality [8,9]. However, the prognostic effect of age was questioned since older patients were less likely to receive standard of care than young patients [10]. A large cohort study from Denmark showed that chronological age has no influence on survival when the older patients receive standard treatment [11].

The treatment characteristics of older patients with OC were different from those of young patients. The standard of care was adopted in 83% of the young group as compared to a much lower 45% of the older group [10]. Older patients were less likely to receive aggressive surgery, and the rate of R0 cytoreduction was low [10]. Second, the rate of discontinuation of adjuvant chemotherapy was higher in older patients than in young patients Ref. [12], and more older patients received monotherapy as adjuvant chemotherapy [13]. Third, most of older patients were excluded from clinical trials [9,14,15]. Conclusions drawn from these studies need further validation among older patients with cancer.

Due to heterogeneity in the treatment for the younger and older patients, the results of the simple comparison of prognosis between the two groups are not solid. In addition, in previous studies [9,16], the age span of the young group (as a control group) was relatively large when compared to that of the older group. Heterogeneity within the young group also leads to conflicting results among different studies. Therefore, we used propensity score matching, a statistical method, to reduce the confounding effect of chronological age, fragility, and treatment disparities.

The objective of this two-center retrospective cohort study was to investigate the effect of different treatment modalities on the prognosis of patients aged 60 years or older with OC in China. Our findings may help to find a more suitable treatment method for older patients.

## 2. Materials and Methods

### 2.1. Study Cohort

All OC patients treated with cytoreductive surgery and adjuvant chemotherapy between 2011 and 2019 were reviewed. The cohort was divided into two different groups by age at surgery, establishing cutoff age values of <70 and ≥70 years.

Patients included in the study were required to have undergone cytoreductive surgery followed by platinum-based adjuvant chemotherapy in either Cancer Hospital Chinese Academy of Medical Science or Beijing Hospital for treating high-grade epithelial carcinoma. Patients with tumors of low-grade carcinoma, nonepithelial carcinoma, and those who received no surgery or palliative surgery were excluded.

All patients included in our study had an Eastern Cooperative Oncology Group (ECOG) performance status (PS) of 0–2. In total, 324 patients with newly diagnosed International Federation of Gynecology and Obstetrics (FIGO) stage IC to IVB ovarian cancer met the inclusion criteria. All clinical data, including cohort demographic information and treatment modalities, were extracted from the electronic records in the two hospitals.

### 2.2. Surgery

A newly diagnosed OC patient received primary debulking surgery (PDS) when complete resection of all visible tumors was feasible as judged by at least two gynecological oncologists using enhanced CT or MRI. However, there was no consensus on the selection criteria for PDS. All patients staged FIGO I–II by imaging received PDS. Patients who were not considered candidates for PDS received two to four cycles of neo-adjuvant chemotherapy (NAC) followed by interval debulking surgery (IDS).

The performance status defined by the ECOG, and the American Society of Anesthesiologist (ASA) grade before surgery were recorded. The Charlson Comorbidity Index (CCI) is widely used as an assessment tool for the severity of comorbidities [17]. Therefore, by retrospectively retrieving 17 medical conditions, the CCI scores were obtained.

Tumor dissemination was classified into three groups, as previously defined by Aletti [18]. Low tumor dissemination (LTD) referred to pelvic lymph node and/or omentum involvement without carcinomatosis. High tumor dissemination (HTD) referred to tumor nodules (diameter > 4 cm) on the surface of the diaphragm and on the mesentery or the presence of liver metastasis. Intermediate tumor dissemination (ITD) referred to those in-between. The extent of the surgical procedure was evaluated using the surgical complexity score (SCS) [19].

Staging was determined using the FIGO staging system. The completeness of cytoreduction was determined based on the volume of residual tumor after surgery, with R0 defined as no visible evidence of tumor. Optimal cytoreduction was defined as a residual tumor less than 1 cm in diameter.

### 2.3. Systemic Chemotherapy and Chemotherapy-Related Toxicity

The PDS group received four to eight cycles of adjuvant platinum-based chemotherapy. Chemotherapy regimens were paclitaxel (150–175 mg/m^2^)/carboplatin (AUC 4–5), paclitaxel (150–175 mg/m^2^)/nedaplatin (70–80 mg/kg), and single agent chemotherapy (carboplatin). The hemoglobin and albumin levels before postoperative adjuvant chemotherapy were recorded. The IDS group received two to four cycles of platinum-based chemotherapy before cytoreductive surgery and zero to five additional cycles of chemotherapy after surgery. Completion of adjuvant chemotherapy was defined as receiving ≥6 cycles of postoperative chemotherapy in PDS or six cycles of chemotherapy in total in IDS.

The biochemical data comprised postoperative hemoglobin, creatinine, and albumin. The interval between cytoreductive surgery and biochemical test was over 2 weeks. The value of serum albumin before the first cycle of post-operative adjuvant chemotherapy was recorded, and it was used as the biochemical marker of nutritional status. The risk of severe chemotherapy-related toxicity was evaluated by Cancer Aging Research Group (CARG) scores [20]. Chemotherapy-related toxicity was graded according to the Common Terminology Criteria for Adverse Events (CTCAE, version 4.0).

### 2.4. Prognosis and Follow-Up Evaluation

The clinical follow-up evaluation included physical and radiographic examination as well as tumor marker assessment. Recurrence was defined as unequivocal radiologic evidence of progression of residual tumor or emerging new tumor lesion with or without elevated CA125. PFS was calculated from the date of the last dose of chemotherapy to the date of recurrence or death, which occurred first.

### 2.5. Statistical Analysis

A binary logistic regression analysis was performed to define the risk factors for grade III-IV hematological toxicity, and a multiple logistic regression analysis was performed to define the independent risk factors. The Kaplan–Meier method was used to estimate PFS for patients in the Age < 70 group and the Age ≥ 70 group. A propensity score-matched analysis was performed using a multivariate logistic regression model based on NAC, renal function, surgical complexity score, ascites, systemic lymph node dissection, R0 cytoreduction, FIGO stage, histology, chemotherapy regimen, and completion of adjuvant chemotherapy. Pairs of patients were derived using 1:1 greedy nearest neighbor matching within PS scores of 0.02. This strategy resulted in 81 matched pairs in each group. All analyses were performed using SPSS version 24.0 (SPSS, Chicago, IL, USA). A *p* value less than 0.05 was considered significant.

## 3. Results

### 3.1. Demographics of the Original Cohort

Of the 324 EOC patients reviewed, 138 (42.6%) were aged 70 years and above (the Age ≥ 70 group) with a median age of 73.4 (70.1–82.1) years, and 186 (57.4%) were 60–70 years (the Age < 70 group) with a median age of 64.8 (61.7–69.7) years. The demographics of the two groups are presented in Table 1.

A total of 180 (55.6%) were treated with PDS, and 144 (44.4%) were treated with NAC + IDS. Eighty-six (62.3%) patients in the Age ≥ 70 group and 58 (31.2%) patients in the Age < 70 group received NAC+IDS as the primary treatment (*p* < 0.001). R0 cytoreduction was achieved in 84 (45.2%) patients in the Age ≥ 70 group and 55 (39.9%) patients in the Age < 70 group (*p* = 0.340). The distribution of FIGO stage between the two groups was not significantly different (*p* = 0.228).

There was no significant difference between the Age < 70 group and the Age ≥ 70 group regarding performance status (*p* = 0.062), hypoalbuminemia (3.8% vs. 2.9%, *p* = 0.711) or anemia (31.2% vs. 39.1%, *p* = 0.331). The distribution of ASA grade in the two groups was comparable (*p* = 0.059). However, the renal function of the Age ≥ 70 group was much worse than that of the Age < 70 group (*p* < 0.001). Additionally, the Age ≥ 70 group had higher CCI (*p* = 0.004) and CARG (*p* < 0.001) scores. The Age ≥ 70 group received fewer cycles of adjuvant chemotherapy than the Age < 70 group (median cycles of chemotherapy was 4.64 and 5.72, respectively, *p* = 0.014). The rate of completion of adjuvant chemotherapy in the Age ≥ 70 group was lower than that in the Age < 70 group (68.8% vs. 89.2%, *p* < 0.001).

### 3.2. Development of the Cohort after PSM

Since the distributions of patient characteristics between the two groups were not equal, a propensity score-matched analysis was performed based on NAC, renal function (<60 mg/min/1.73 m^2^), volume of ascites (<200 mL), SCS grades, systemic lymph node dissection, histology, R0 cytoreduction, FIGO stage, completion of chemotherapy, and chemotherapy regimen. The demographics of the cohort after PSM are presented in Table S2.

### 3.3. Prognosis

**Figure 1:**
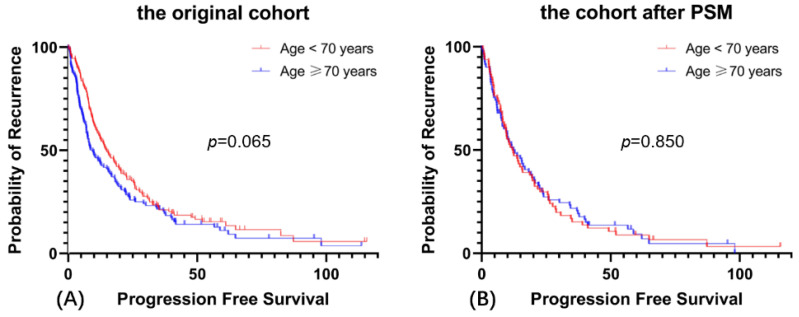
The prognostic analysis of age on PFS. (**A**) Progression-free Survival in the original cohort, (**B**) Progression-free Survival in the cohort after PSM.

At the time of the last follow-up, 264 of 324 women were confirmed to have recurrent disease: 148 in the Age < 70 group and 116 in the Age ≥ 70 group. The median follow-up duration was 24.5 months in the original cohort. The median PFS for the Age < 70 group was 14.8 months, while the median PFS for the Age ≥ 70 group was 9.5 months (*p* = 0.065, Figure 1A). The young patients had longer PFS than the older patients, even though the difference did not reach statistical significance. In the cohort after PSM, there was no significant difference in PFS between these two groups (median PFS = 12.4 and 11.9, *p* = 0.850) as shown in Figure 1B.

**Figure 2:**
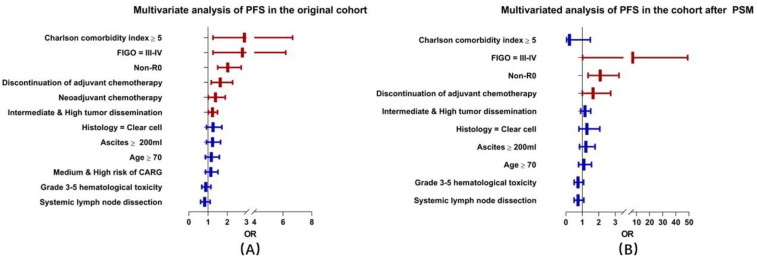
Multivariate analysis of PFS in the original cohort and the cohort after PSM. (**A**) Multivariate analysis of progression-free survival in the original cohort, (**B**) Multivariate analysis of progression-free survival in the cohort after PSM. (The characteristics with *p* < 0.05 are shown in red, while other characteristics with *p* ≥ 0.05 are shown in blue.).

We further investigated the independent factors associated with PFS. In the original cohort, multivariate analysis showed that CCI ≥ 5 (HR = 2.747, 95% CI = 1.245–6.061, *p* = 0.012), NAC (HR = 1.163, 95% CI = 1.048–1.290, *p* = 0.005), intermediate and high tumor dissemination (HR = 1.221, 95% CI = 1.010–1.477, *p* = 0.039), non-R0 cytoreduction (HR = 2.058, 95% CI = 1.534–2.755, *p* < 0.001), FIGO stage III–IV (HR = 2.311, 95% CI = 1.558–3.426, *p* < 0.001), and discontinuation of adjuvant chemotherapy (HR = 1.458, 95% CI = 1.041–2.041, *p* = 0.028) were independent risk factors for shorter PFS. In the cohort after PSM, non-R0 cytoreduction (HR = 2.087, 95% CI = 1.350–3.226, *p* = 0.001), FIGO stage III–IV (HR = 7.051, 95% CI = 1.015–49.01, *p* = 0.048), and discontinuation of adjuvant chemotherapy (HR = 1.653, 95% CI = 1.001–2.725, *p* = 0.049) were independent factors for worse PFS (Figure 2 in red, and Table S1).

**Figure 3:**
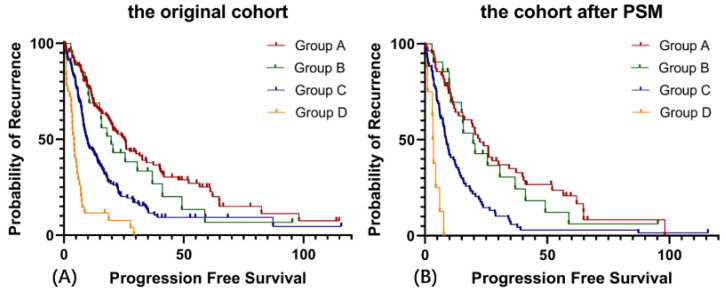
Prognostic analysis of different treatment patterns in both cohorts. (**A**) Progression-free Survival in the original cohort, (**B**) Progression-free Survival in the cohort after PSM. (Group A: Patients received R0 cytoreduction followed by completion of adjuvant chemotherapy. Group B: Patients received R0 cytoreduction followed by discontinuation of adjuvant chemotherapy. Group C: Patients received non-R0 cytoreduction followed by completion of adjuvant chemotherapy. Group D: Patients received non-R0 followed by discontinuation of adjuvant chemotherapy.).

As shown in Figure 3, the patients who underwent R0 cytoreduction with completion of adjuvant chemotherapy (Group A) had the best prognosis, with a median PFS of 25.2 months and 22.4 months, respectively. The PFS of patients who underwent R0 cytoreduction with discontinuation of adjuvant chemotherapy (Group B) was significantly longer than that of patients who underwent non-R0 cytoreduction with completion of adjuvant chemotherapy (Group C, *p* = 0.023 and 0.005, respectively).

Based on the volume of residual tumor (no tumor residue, ≤0.5 cm, 0.5–1 cm, and >1 cm), we further divided patients into four different subgroups. The median PFS of these groups was 24.6, 11.6, 7.3, and 6.8 months (*p* < 0.001) as shown in Figure S1. In the Age ≥ 70 group, the R0 subgroup had the best prognosis, with a median PFS of 30.1 months (*p* < 0.001), while the median PFS of the other three subgroups was 7.1, 7.1, and 5.3 months, respectively. In the Age < 70 group, the R0 subgroup had the best prognosis, with a median PFS of 20.5 months (*p* < 0.001). The subgroup with residual tumor < 0.5 cm (mPFS = 15.8 months) had a better prognosis than those with residual tumor 0.5–1 cm (mPFS = 8.1 months, *p* = 0.008) and >1 cm (mPFS = 8.1 months, *p* = 0.001).

**Figure 4:**
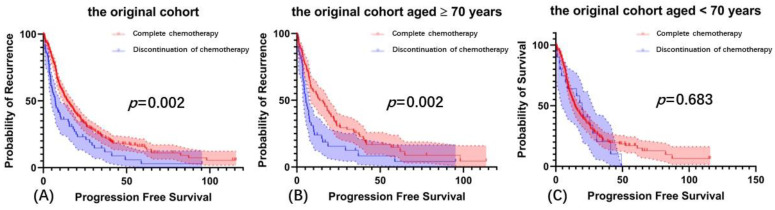
Prognostic analysis of completion of adjuvant chemotherapy on PFS. (**A**) Progression-free survival in the original cohort, (**B**) Progression-free survival in the Age ≥ 70 group, (**C**) Progression-free survival in the Age < 70 group.

Patients who received completion of adjuvant chemotherapy had longer PFS than those who did not (PFS = 14.1 vs. 7.1, *p* = 0.002, Figure 4A). Among patients aged ≥70 years, discontinuation of adjuvant chemotherapy significantly affected PFS. The R0 rates in the discontinuation of adjuvant chemotherapy group and completion of adjuvant chemotherapy group were 27.9% and 45.3%, respectively (*p* = 0.054, Figure 4B). Among patients aged <70 years, the difference in PFS between the completion of adjuvant chemotherapy group and discontinuation of adjuvant chemotherapy group was not significant (*p* = 0.683, Figure 4C). 

Over 75% of patients in the discontinuation of adjuvant chemotherapy group received R0 cytoreduction, while the R0 rate in the completion of adjuvant chemotherapy group was 41.6% (*p* = 0.005).

### 3.4. Severe Chemotherapy-Related Toxicity

An increased prevalence of severe toxicity (grade III–IV) was observed (45.6% vs. 34.2%, *p* = 0.040) in the Age ≥ 70 group. Univariate analysis in the original cohort showed that the risk factors were age ≥ 70 years and albumin level < 40 g/L (Table 2). Multivariate analysis in the original cohort showed that the only independent risk factor was albumin level < 40 g/L (*p* < 0.001, OR = 2.860, 95% CI = 1.746–4.684). We further validated the risk factors in the cohort after PSM. In multivariate analysis, the independent risk factors for severe hematological toxicity were age ≥ 70 years (*p* = 0.008, OR = 2.639, 95% CI = 1.291–5.395) and albumin level < 40 g/L (*p* = 0.018, OR = 2.441, 95% CI = 1.168–5.100).

### 3.5. Cause of Discontinuation of Adjuvant Chemotherapy

Of the 324 patients reviewed, discontinuation of adjuvant chemotherapy occurred in 63 (19.4%) patients. We investigated the independent risk factors of discontinuation of adjuvant chemotherapy. In the original cohort, poor renal function and NAC were associated with discontinuation of adjuvant chemotherapy. In the cohort after PSM, the only independent risk factor for discontinuation of adjuvant chemotherapy was poor renal function (HR = 5.128, 95% CI = 1.789–14.71, *p* = 0.002) as shown in Table 3.

## 4. Discussion

In this propensity score-matched study from two national centers in China, we found that patients aged over 70 years had similar PFS to patients aged 60–70 years. Different treatment characteristics (non-R0 cytoreduction or discontinuation of adjuvant chemotherapy) were associated with worse progression-free survival. To the best of our knowledge, very few studies are available on how chronological age and different treatment modalities affect prognosis in two matched cohorts. In addition, our results highlight the importance of serum albumin (<40 g/L), tested immediately before the first cycle of adjuvant chemotherapy, as a clinical indicator of severe chemotherapy-related toxicities.

One of the main goals was to evaluate the effect of chronological age on the prognosis of the older patients. Generally, high chronological age is seen as a poor prognostic factor. In 2022, Shim reviewed 21,446 women between 2006 and 2016 using the database of the Korea Central Cancer Registry. The results showed that age > 65 years was associated with a higher mortality rate [21]. Another study conducted by Joueidi et al. retrospectively reviewed 615 patients aged <65 years and 364 patients aged over 70 years. 

The 3-year survival was worse for older patients, while the 3-year disease-free survival was comparable among groups with different ages [22]. The older patients included in Joueidi’s study received less radical surgery, had more residual tumors, and received fewer cycles of adjuvant chemotherapy. As a result, the survival of older patients may be underestimated. We compared the prognosis of patients aged <70 years and ≥70 years in the cohort after PSM to control possible confounding variables. The results indicated that high chronological age may not be associated with shorter PFS.

Cytoreduction with no visible tumor has long been recognized as one of the most important prognostic factors for patients with OC [23,24,25]. Piedimonte et al. retrospectively analyzed 248 women aged ≥70 years to evaluate the significance of R0 cytoreduction on prognosis [26]. Sixty-eight patients received R0 cytoreduction in the PDS and IDS groups. The patients who received PDS with no residual tumor had the best prognosis, with a median PFS of 23.5 months. 

In our study, the rate of R0 cytoreduction in the PDS and IDS groups altogether was 39.9% in the Age ≥ 70 group, with a median PFS of 24.6 months. The PFS of the residual tumor ≤ 0.5 cm, ≤1 cm, and >1 cm groups were comparable, and they were all inferior to that of the R0 group (*p* < 0.001). In multivariate analysis, R0 cytoreduction was an independent prognostic factor in the cohort after PSM. R0 cytoreduction served as an important prognostic factor for older patients with ovarian cancer.

R0 cytoreduction always involves more radical surgical procedures, including peritoneum stripping, bowel resection, splenectomy, and liver resections. The debilitated state of older patients leads to severe postoperative morbidities [18,27] and consequently leads to delay or discontinuation of adjuvant chemotherapy, which may negate the benefit of R0 cytoreduction. A more recent study indicated that increased 90-day morbidity leads to a lower 3-year survival [28]. A commonly raised argument in that regard questions whether optimal cytoreduction with residual tumor < 1 cm followed by adjuvant chemotherapy would be more suitable for older patients with high fragility. 

Our study contradicted this assumption. Patients who received R0 cytoreduction and completion of adjuvant chemotherapy had the best prognosis. The efficacy of non-R0 cytoreduction with completion of adjuvant chemotherapy was significantly inferior to R0 cytoreduction with discontinuation of adjuvant chemotherapy. The efficacy of R0 cytoreduction seemed to outweigh completion of chemotherapy. Maximum surgical effort with no residual tumor, other than optimal debulking with residual tumor < 1 cm, should be a preferred treatment modality for older patients.

Discontinuation of adjuvant chemotherapy was believed to have a negative influence on prognosis [16]. Severe chemotherapy-related toxicity is one of the most common reasons for discontinuation of adjuvant chemotherapy [29,30]. The relationship between chronological age and chemotherapy toxicity is contradictory. 

HAY et al. prospectively evaluated eighty patients with ovarian cancer and found that age was not associated with tolerance to chemotherapy [31]. A case–control study conducted by Amadio showed that the incidence of severe toxicity in the young and older groups who received platinum-based chemotherapy and bevacizumab was comparable [32]. 

In our study, multivariate analysis showed that age ≥ 70 years and albumin level < 40 g/L were predictors of severe chemotherapy-related toxicity. There were several reasons why age ≥ 70 years was found to be an independent risk factor for severe chemotherapy toxicity in our study. First, all patients recruited in this study underwent cytoreductive surgery. Tolerance to chemotherapy might be affected. Second, the results of the multivariate analysis might vary with different cutoff values of age since there is no universal cutoff value for the older cohort [33].

Severe chemotherapy-related toxicities may be associated with low serum albumin level. In the bloodstream, paclitaxel was delivered to tumor lesion in an albumin-bound form. Binding paclitaxel to albumin decreased chemotherapy-related toxicities and increased the distribution of paclitaxel in tumors [34]. A prospective study on patients who were treated with paclitaxel-cisplatin chemotherapy showed that chemotherapy-related toxicity was significantly associated with hypoalbuminemia (<31 g/L, 35 g/L, or <38 g/L) [35]. 

Our results highlighted that the risk of severe hematological toxicity significantly increased when serum albumin dropped below 40 g/L (HR = 2.860, 95% CI = 1.746–4.684, *p* < 0.001). After adjusting for treatment characteristics, serum albumin <40 g/L was still a strong indicator of severe chemotherapy-related toxicity (HR = 2.441, 95% CI = 1.168–5.100, *p* < 0.018). The association between albumin levels and chemotherapy toxicity has seldom been reported and requires further investigation. Serum albumin could be used not only as an indicator of nutritional status but also as an indicator of severe chemotherapy-related toxicity.

There were certain limitations in this study. Because of the retrospective nature of the study, selection bias was unavoidable. We selected older patients who underwent surgery and excluded those who could not undergo surgery. As a result, the patients included had relatively good performance status and fewer comorbidities. The prognosis of older patients with high fragility needs further evaluation. Moreover, there was heterogeneity of treatment between the two groups of selected patients. However, we used the propensity score matching method to minimize the confounding effect. 

Another limitation of this study might be the use of progression-free survival (PFS) instead of overall survival (OS). In our opinion, PFS or cancer-specific mortality may be more appropriate than OS for evaluating the prognosis of older patients because the life expectancy of the older cohort was shorter than that of the younger cohort. Nevertheless, most patients included were before maintenance therapy of the Poly (ADP-ribose) polymerase (PARP) inhibitor was approved in patients with BRCA mutations in China. The adoption of genetic testing and maintenance therapy was insufficient.

Despite the limitations mentioned above, one of the strengths of our study is the establishment of a case–control cohort with a relatively large cohort to investigate the effect of chronological age and treatment modalities on prognosis. The confounding effect between the two groups was significantly reduced. Another strength of our study is the large proportion of older patient (138, 42.6%) data collected from two national centers in China.

## 5. Conclusions

In conclusion, older patients with OC benefit most from cytoreduction with no residual tumor followed by adjuvant chemotherapy with no discontinuation. Both non-R0 cytoreduction and the discontinuation of adjuvant chemotherapy may independently affect the efficacy of treatment. Alterations to standard treatment due to high chronological age should be avoided. 

Taking necessary precautions for patients with a high risk of discontinuing adjuvant chemotherapy may further improve the prognosis of older cohorts receiving cytoreduction.

## Figures and Tables

**Table 1:** Demographic information of the original cohort.

Characteristics		N (%)	Groups, *n* (%)		*p* Value
			Age < 70 (*n* = 186)	Age ≥ 70 (*n* = 138)	
Age (years)	Median ± SD	68.5 ± 5.3	64.8 ± 3.1	73.4 ± 3.3	<0.001
BMI (kg/m^2^)	Median ± SD	24.4 ± 3.3	24.5 ± 3.4	24.3 ± 3.0	0.909
BRCA testing	No	248 (76.5)	135 (72.6)	113 (81.9)	0.051
	Yes	76 (23.5)	51 (27.4)	25 (18.1)	
BRCA 1/2 mutation	negative	60 (78.9)	40 (78.4)	20 (80.0)	0.875
	positive	16 (21.1)	11 (21.6)	5 (20.0)	
Performance status	0	253 (79.3)	151 (83.4)	102 (73.9)	0.062
	1	65 (20.4)	29 (16.0)	36 (26.1)	
	2	1 (0.3)	1 (0.6)	0 (0.0)	
Hemoglobin (g/L)	≥110	202 (62.3)	122 (65.6)	80 (58.0)	0.331
	<110	112 (34.6)	58 (31.2)	54 (39.1)	
	unknown	10 (3.1)	6 (3.2)	4 (2.9)	
Albumin (g/L)	>40	144 (47.7)	90 (51.4)	54 (42.5)	0.126
	≤40	158 (52.3)	85 (48.6)	73 (57.5)	
	unknown	22 (6.8)	11 (5.9)	11 (8.0)	
Renal function	>90	84 (26.5)	65 (35.9)	19 (14.0)	<0.001
(mL/min*1.73 m^2^)	60–90	186 (58.7)	105 (58.0)	81 (59.6)	
	30–60	45 (14.2)	11 (6.1)	34 (25.0)	
	15–30	2 (0.6)	0 (0.0)	2 (1.5)	
	<15	0 (0.0)	0 (0.0)	0 (0.0)	
Neoadjuvant chemotherapy	No	180 (55.6)	128 (68.8)	52 (37.7)	<0.001
	Yes	144 (44.4)	58 (31.2)	86 (62.3)	
ASA	1	20 (6.2)	14 (7.5)	6 (4.3)	0.059
	2	238 (73.5)	142 (76.3)	96 (69.6)	
	3	66 (20.4)	30 (16.1)	36 (26.1)	
Ascites	<200 mL	202 (62.3)	107 (57.5)	95 (68.8)	0.038
	≥200 mL	122 (37.7)	79 (42.5)	43 (31.2)	
Tumor dissemination	Low	108 (33.3)	62 (33.3)	46 (33.3)	0.999
	Intermediate	143 (44.1)	82 (44.1)	61 (44.2)	
	High	73 (22.5)	42 (22.6)	31 (22.5)	
Surgical complexity score	1	224 (75.3)	130 (69.9)	114 (82.6)	0.009
	2	80 (24.7)	56 (30.1)	24 (17.4)	
	3	0 (0.0)	0 (0.0)	0 (0.0)	
Systemic lymph node dissection	No	105 (60.2)	96 (51.6)	99 (71.7)	<0.001
	Yes	129 (39.8)	90 (48.4)	39 (28.3)	
FIGO stage	I	13 (4.0)	10 (5.4)	3 (2.2)	0.228
	II	32 (9.9)	21 (11.3)	11 (8.0)	
	III	229 (70.7)	124 (66.7)	105 (76.1)	
	IV	50 (15.4)	31 (16.7)	19 (13.8)	
Histology	HGSC	297 (91.7)	164 (88.2)	133 (96.4)	0.030
	EC	11 (3.4)	9 (4.8)	2 (1.4)	
	Clear cell	16 (4.9)	13 (7.0)	3 (2.2)	
R0 cytoreduction	No	185 (57.1)	102 (54.8)	83 (60.1)	0.340
	Yes	139 (42.9)	84 (45.2)	55 (39.9)	
CARG	low risk	209 (64.5)	157 (84.4)	52 (37.7)	<0.001
	medium risk	113 (34.9)	29 (15.6)	84 (60.9)	
	high risk	2 (0.6)	0 (0.0)	2 (1.4)	
Cycles of chemotherapy	Median ± SD	5.26 ± 1.84	5.72 ± 1.67	4.64 ± 1.85	0.014
Completion of adjuvant chemotherapy	No	63 (19.4)	20 (10.8)	43 (31.2)	<0.001
	Yes	261 (80.6)	166 (89.2)	95 (68.8)	
Chemotherapy	none	2 (0.6)	0 (0.0)	2 (1.4)	0.008
regimen	monotherapy	5 (1.5)	0 (0.0)	5 (3.6)	
	double therapy	317 (97.9)	186 (1.0)	131 (95.0)	
Hematologic toxicity	I–II	195 (60.9)	121 (65.8)	74 (54.4)	0.040
	III–IV	125 (39.1)	63 (34.2)	62 (45.6)	
Clinical trial	No	317 (97.8)	182 (97.8)	135 (97.8)	0.989
	Yes	7 (2.2)	4 (2.2)	3 (2.2)	
Charlson comorbidity index	mild	0 (0.0)	0 (0.0)	0 (0.0)	0.004
	moderate	35 (10.8)	28 (15.1)	7 (5.1)	
	sever	289 (89.2)	158 (84.9)	131 (94.9)	

HGSC, high-grade serous carcinoma; and EC, endometroid carcinoma.

**Table 2:** Risk factors for III–V chemotherapy-related hematologic toxicity.

Characteristics		The Original Cohort			The Cohort after PSM		
		Univariate	Multivariate Analysis		Univariate	Multivariate Analysis	
		*p*	OR (95% CI)	*p*	*p*	OR (95% CI)	*p*
Age	<70	0.040	1	0.083	0.010	1	0.008
	≥70		1.602 (0.940–2.729)			2.639 (1.291–5.395)	
Renal function	<60	0.957	1	0.608	0.686	1	0.635
	>60		0.824 (0.393–1.727)			1.330 (0.409–4.323)	
Albumin (g/L)	≥40	<0.001	1	<0.001	0.010	1	0.018
	<40		2.860 (1.746–4.684)			2.441 (1.168–5.100)	
Surgical complexity score	1	0.085	1	0.093	0.313	1	0.561
	2–3		1.614 (0.923–2.821)			1.272 (0.565–2.863)	
Completion of adjuvant chemotherapy	No	0.607	1	0.886	0.149	1	0.208
	Yes		1.048 (0.553–1.984)			0.554 (0.221–1.389)	
Chemotherapy regimen	monotherapy	0.061	1	0.088	N/A	N/A	N/A
	double therapy		7.355 (0.745–72.598)				

N/A, not applicable.

**Table 3:** Caused of discontinuation of adjuvant chemotherapy in the original cohort and the cohort after PSM.

Characteristics		The Original Cohort			The Cohort after PSM		
		Univariate	Multivariate		Univariate	Multivariate	
		*p* value	OR (95% CI)	*p* value	*p* value	OR (95% CI)	*p* value
Age	<70	<0.001	1	0.182	0.539	1	0.497
	≥70		1.634 (0.795–3.356)			1.441 (0.556–3.733)	
Charlson comorbidity index	3–4	0.098	1	0.991	0.380	1	0.952
	≥5		1.008 (0.254–4.003)			0.947 (0.165–5.443)	
Cancer Aging Research Group scores	Low risk	0.001	1	0.666	0.460	1	0.575
	Medium risk		0.858 (0.428–1.719)			0.756 (0.285–2.006)	
ASA	1–2	0.005	1	0.061	0.283	1	0.404
	3		1.957 (0.968–3.953)			0.640 (0.224–1.827)	
Renal function	>60	<0.0001	1	0.010	0.001	1	0.002
	<60		2.717 (1.266–5.814)			5.128 (1.789–14.71)	
Neoadjuvant chemotherapy	No	<0.0001	3.367 (1.626–6.944)	0.001	0.041	2.326 (0.859–6.289)	0.097
	Yes						
Surgical complexity score	1	0.141			0.445		
	2–3						
Ascites	<200 mL	0.027	1	0.089	0.174		
	≥200 mL		1.893 (0.908–3.947)				
Hematologic toxicity	0–2	0.606			0.150		
	3–4						
Chemotherapy	monotherapy	0.247			N/A		
regimen	double therapy						

N/A, not applicable.

## Data Availability

The data that support the findings of this study are available from the corresponding author, L.W., upon reasonable request.

## Supplementary Materials

The following supporting information can be downloaded at: https://www.mdpi.com/article/10.3390/cancers14153655/s1, Table S1: Univariate analysis and multivariate analysis of PFS. Table S2: Demographic information of the cohort after PSM. Figure S1: Prognostic analysis of residual tumor on PFS.
