# Supplementary material for: Differences in Treatment Modalities and Prognosis of Elderly Patients with Ovarian Cancer: A Two-Center Propensity Score-Matched Study
Source: Cancers (Basel). 2022 Jul 27;14(15):3655. doi: 10.3390/cancers14153655 (PMC9367487; doi:10.3390/cancers14153655)
Supplement: Supplementary file 1 [file cancers-14-03655-s001.zip › cancers-1772016-supplementary.pdf]

cancers

MDPI

Supplementary Materials

Differences in Treatment Modalities and Prognosis of Elderly Patients with Ovarian Cancer: A Two-Center Propensity Score-Matched Study

Yuxi Zhao, Jing Zuo, Ning Li, Rongshou Zheng, Guanwen Yuan, Guihua Shen* and Lingying Wu*

Table S1. Univariate analysis and multivariate analysis of PFS.

Characteristics		The Original Cohort				The Cohort after PSM			
		Univariate		Multivariate		Univariate		Multivariate	
		OR (95% CI)	*p*	OR (95% CI)	*p*	OR (95% CI)	*p*	OR (95% CI)	p
Age	<70	**1**	**0.068**	1	0.301	1	0.850	1	0.741
	≥70	**1.256 (0.985–1.603)**		1.174 (0.866–1.591)		0.969 (0.698–1.345)		1.096 (0.771–1.558)	
CCI	3–4	**1**	**0.011**	**1**	**0.012**	**1**	**0.008**	1	0.120
	≥5	**1.705 (1.128–2.577)**		**2.899 (1.259–6.667)**		**2.172 (1.221–3.865)**		0.219 (0.032–1.487)	
CARG	Low risk	**1**	**0.021**	1	0.341	1	0.304		
	Medium risk	**1.327 (1.044–1.687)**		1.146 (0.866–1.516)		1.188 (0.855–1.651)			
ASA	1	1				1			
	2	1.288 (0.755–2.195)	0.353			0.872 (0.447–1.701)	0.688		
	3	0.859 (0.636–1.160)	0.321			0.739 (0.480–1.139)	0.170		
NAC	No	**1**	**0.001**	**1**	**0.040**	1	0.408		
	Yes	**1.509 (1.183–1.924)**		**1.388 (1.015–1.897)**		1.152 (0.824–1.608)			
Renal function	<60	1	0.881			1	0.064		
	>60	0.973 (0.681–1.391)				0.582 (0.329–1.031)			
Hb	≥100	1	0.056			1	0.502		
	<100	1.166 (0.996–1.366)				1.093 (0.843–1.417)			
Alb	<40	1	0.269			1	0.828		
	≥40	1.154 (0.895–1.487)				1.039 (0.737–1.465)			
SCS	1	1	0.189			1	0.976		
	2–3	0.829 (0.626–1.097)				0.994 (0.673–1.469)			
Ascites	<200	**1**	**0.001**	1	0.115	**1**	**0.001**	1	0.316
	≥200	**1.553 (1.210–1.993)**		1.236 (0.923–1.655)		**1.809 (1.281–2.556)**		1.215 (0.830–1.779)	
TD	LTD	**1**	**<0.001**	**1**	**0.036**	**1**	**<0.001**	1	0.246
	ITD & HTD	**1.533 (1.313–1.790)**		**1.231 (1.014–1.494)**		**1.498 (1.219–1.840)**		1.165 (0.900–1.509)	
SLND	No	**1**	**<0.001**	1	0.208	**1**	**0.012**	1	0.129
	Yes	**0.619 (0.481–0.797)**		0.827 (0.615–1.112)		**1.570 (1.104–2.237)**		0.740 (0.501–1.092)	
R0	Yes	**1**	**<0.001**	**1**	**<0.001**	**1**	**<0.001**	**1**	**0.001**

	No	**2.273 (1.760–2.924)**		**2.028 (1.506–2.732)**		**2.421 (1.718–3.413)**		**2.087 (1.350–3.226)**	
FIGO stage	I-II	**1**	**<0.001**	**1**	**0.011**	**1**	**0.001**	**1**	**0.048**
	III-IV	**2.311 (1.558–3.426)**		**2.795 (1.261–6.194)**		**2.731 (1.535–4.858)**		**7.051 (1.015–49.01)**	
Histology	HGSC	1	0.387	1	0.145	1	0.563	1	0.309
	Clear cell	0.883 (0.667–1.170)		1.261 (0.923–1.722)		0.883 (0.579–1.346)		1.281 (0.795–2.064)	
HT	0–2	1	0.249	1	0.357	1	0.093	1	0.116
	3–4	0.863 (0.672–1.108)		0.883 (0.677–1.151)		0.741 (0.523–1.051)		0.736 (0.502–1.079)	
CAC	Yes	**1**	**0.002**	**1**	**0.004**	1	0.922	**1**	**0.049**
	No	**1.597 (1.183–2.160)**		**1.634 (1.170–2.283)**		1.021 (0.663–1.575)		**1.653 (1.001–2.725)**	

Hb, hemoglobin; Alb, albumin; SLND, systemic lymph node dissection; CAC, completion of adjuvant chemotherapy; HT, hematologic toxicity; TD, tumor dissemination.

Table S2. Demographic information of the cohort after PSM.

Characteristics		n (%)	Groups, n (%)		*p* Value
			Age<70 (n = 81)	Age≥70 (n = 81)	
NAC	No	73(45.1)	37 (45.7)	36 (44.4)	0.875
	Yes	89(54.9)	44 (54.3)	45 (55.6)	
Renal function	≥60	143(88.3)	71(87.7)	72(88.9)	0.807
	<60	19(11.7)	10(12.3)	9(11.1)	
Ascites	<200 ml	105(64.8)	53 (65.4)	52 (64.2)	0.869
	≥200 ml	57(35.2)	28 (34.6)	29 (35.8)	
SCS	1	126	65 (80.2)	61 (75.3)	0.450
	2	36	16 (19.8)	20 (24.7)	
	3	0	0(0.0)	0(0.0)	
SLND	No	103(63.6)	49 (60.5)	54 (66.7)	0.414
	Yes	59(36.4)	32 (39.5)	27 (33.3)	
Histology	HGSC	146(90.1)	70(86.4)	76 (93.8)	0.205
	EC	9(5.6)	7 (8.6)	2 (2.5)	
	Clear cell	7(4.3)	4(4.9)	3(3.7)	
R0 cytoreduction	Yes	86 (53.1)	43 (53.1)	43 (53.1)	1.000
	No	76 (46.9)	38 (46.9))	38 (46.9))	
FIGO stage	I -II	21 (13.0)	13 (16.0)	8 (9.9)	0.242
	III-IV	141 (87.0)	68 (84.0)	73 (90.1)	
CAC	No	29 (17.9)	16 (19.8)	13 (16.0)	0.539
	Yes	133 (82.1)	65 (80.2)	68 (84.0)	
Chemotherapy regimen	None	0 (0.0)	0（0.0）	0（0.0）	1.000
	monotherapy	0 (0.0)	0（0.0）	0（0.0）	
	double therapy	162 (100)	81 (100)	81 (100)	

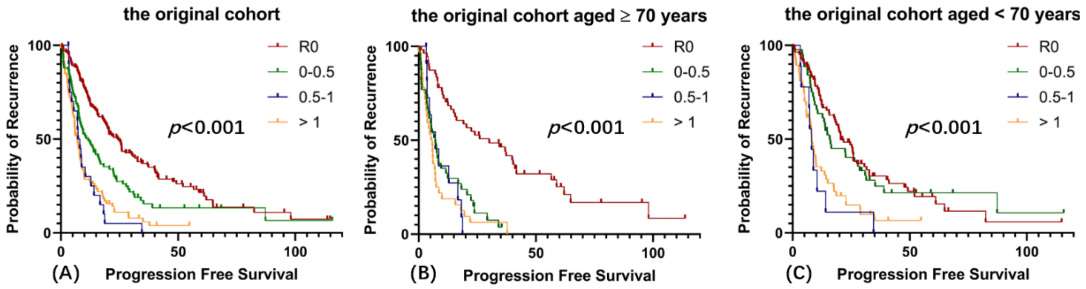

Figure S1. Prognostic analysis of residual tumor on PFS. (**A**) Progression-free survival in the original cohort, (**B**) Progression-free survival in the Age ≥ 70 group, (**C**) Progression-free survival in the Age < 70 group.
